# STIM1 Positively Regulates the Ca^2+^ Release Activity of the Inositol 1,4,5-Trisphosphate Receptor in Bovine Aortic Endothelial Cells

**DOI:** 10.1371/journal.pone.0114718

**Published:** 2014-12-15

**Authors:** Éric Béliveau, Vincent Lessard, Gaétan Guillemette

**Affiliations:** Department of Pharmacology, Faculty of Medicine and Health Sciences, Université de Sherbrooke, Sherbrooke, Québec, Canada, J1H 5N4; The University of Tokyo, Japan

## Abstract

The endothelium is actively involved in many functions of the cardiovascular system, such as the modulation of arterial pressure and the maintenance of blood flow. These functions require a great versatility of the intracellular Ca^2+^ signaling that resides in the fact that different signals can be encoded by varying the frequency and the amplitude of the Ca^2+^ response. Cells use both extracellular and intracellular Ca^2+^ pools to modulate the intracellular Ca^2+^ concentration. In non-excitable cells, the inositol 1,4,5-trisphosphate receptor (IP_3_R), located on the endoplasmic reticulum (ER), is responsible for the release of Ca^2+^ from the intracellular store. The proteins STIM1 and STIM2 are also located on the ER and they are involved in the activation of a store-operated Ca^2+^ entry (SOCE). Due to their Ca^2+^ sensor property and their close proximity with IP_3_Rs on the ER, STIMs could modulate the activity of IP_3_R. In this study, we showed that STIM1 and STIM2 are expressed in bovine aortic endothelial cells and they both interact with IP_3_R. While STIM2 appears to play a minor role, STIM1 plays an important role in the regulation of agonist-induced Ca^2+^ mobilization in BAECs by a positive effect on both the SOCE and the IP_3_R-dependent Ca^2+^ release.

## Introduction

The endothelium is the monolayer of cells lining the interior of blood vessels. The endothelial cells that constitute this monolayer are actively involved in many functions of the cardiovascular system, including the regulation of immune responses, the adjustment of blood-tissue permeability, the repair of blood vessels, the modulation of arterial pressure, and the maintenance of blood flow [1]. Ca^2+^ is a highly versatile second messenger that plays a key role in the regulation of many cellular processes, including secretion, contraction, proliferation, motility, gene expression and cell death [2]. As in other tissues, Ca^2+^ plays an important role in the endothelium. The versatility of Ca^2+^ signaling resides in the fact that different signals can be encoded spatio-temporally by varying the frequency and the amplitude of the Ca^2+^ response [3]. Cells use both extracellular and intracellular Ca^2+^ pools to modulate the intracellular Ca^2+^ concentration. In non-excitable cells, the inositol 1,4,5-trisphosphate receptor (IP_3_R) is responsible for the release of Ca^2+^ from the endoplasmic reticulum (ER), the main intracellular Ca^2+^ store by which the concentration of cytosolic Ca^2+^ is modulated [4]. Three IP_3_R subtypes have been identified to date (IP_3_R-1, IP_3_R-2, and IP_3_R-3) and they associate into tetramers to form functional Ca^2+^ selective ligand-gated channels [2]. IP_3_R is activated by signaling cascades that generate IP_3_. Briefly, an extracellular agonist binds to its specific receptor, which activates phospholipase C (PLC) via a G-protein or a tyrosine kinase activity. PLC then catalyzes the cleavage of phosphatidylinositol 4,5-bisphosphate into diacylglycerol and IP_3_, which diffuses into the cytosol and activates IP_3_R, its receptor/channel [5].

As the Ca^2+^ level in the ER declines, a mechanism of Ca^2+^ entry through the plasma membrane is activated. This “store-operated Ca^2+^ entry” (SOCE) serves to sustain the Ca^2+^ response and to restore the Ca^2+^ level in the ER [6]. The proteins STIM1 and STIM2 (stromal interaction molecule), localized in the membrane of the ER, have recently been identified as Ca^2+^ sensors that, at low luminal Ca^2+^ concentration, activate plasma membrane Ca^2+^ channels members of the Orai or TRPC families [6]–[8].

In endothelial cells, STIM1 has been identified as a crucial component of SOCE and consequently, it is involved in specialized functions that depend on SOCE such as NO production, cell proliferation and *in vitro* VEGF-induced tubulogenesis [9]–[14]. IP_3_R-dependent Ca^2+^ release and SOCE activity both contribute to shape the agonist-induced Ca^2+^ response [15]. The facts that STIMs are sensors of Ca^2+^ in the ER, that they also control the activity of Ca^2+^ channels and that they are located in the ER, where IP_3_Rs also are, make them good candidates to modulate the IP_3_R activity. However, little attention has been given to the potential role of STIMs on IP_3_R-dependent Ca^2+^ release. In this study, we showed that STIM1 and STIM2 are expressed in bovine aortic endothelial cells (BAEC) and participate to SOCE. Most importantly, we showed that STIMs interact with IP_3_R-1 and that the knockdown of STIM1, but not that of STIM2, dampens the IP_3_R-dependent Ca^2+^ release in BAECs.

## Materials and Methods

### Materials

Dulbecco's modified Eagle's medium (DMEM), fetal bovine serum (FBS), and penicillin-streptomycin-glutamine were from Gibco Life Technologies (Gaithersburg, MD, USA). Fura-2/AM was from Calbiochem (San Diego, CA, USA). Anti-IP_3_R-1 antibody was from ABR Affinity Bioreagents (Golden, CO). Anti-STIM1 antibody was from Millipore (Temecula, CA) and anti-STIM2 antibody was from Abcam (Cambridge, MA). Protein AG-Agarose beads and anti-HA probe antibody were from Santa Cruz Technology (Santa Cruz, CA). siRNAs against bovine STIM1 and STIM2, and siRNA control #3 were from Dharmacon, Inc. (Lafayette, CO). LipofectAMINE 2000 transfection reagent was from Invitrogen (Burlington, ON, Canada). ATP and BK were from Sigma-Aldrich (Oakville, ON, Canada).

### Cell cultures

Bovine thoracic aortas from calves 8–10 months of age were obtained from a nearby slaughterhouse (Viandes Giroux, East Angus, QC). BAECs were isolated and characterized as previously described [16]. The cells were maintained in low-glucose DMEM containing 2 mM L-glutamine, 10% FBS, 100 U/ml penicillin, and 100 µg/ml streptomycin at 37°C in a humidified atmosphere containing 5% CO_2_. They were used between the 5th and 20th passages. Experiments were approved by the “Comité facultaire de protection des animaux” of the Faculty of Medicine and Health Sciences of the Université de Sherbrooke.

### Immunoprecipitation and Western blotting

Cells were washed twice with phosphate-buffered saline (137 mM NaCl, 2.8 mM KCl, 1.5 mM KH_2_PO_4_, 8 mM Na_2_HPO_4,_ pH 7.4) and solubilized for 30 min on ice in lysis buffer (50 mM Tris–HCl, pH 7.4, 150 mM NaCl, 1% Triton X-100, 1 mM EDTA, and Complete protease inhibitor cocktail). The lysates were clarified by centrifugation at 10 000× *g* for 10 min. For the immunoprecipitation studies, identical amounts of protein from each sample were incubated overnight at 4°C with 5 µg/ml of a specific antibody. The immune complexes were collected by incubating the mixtures with 50 µl (50% suspension) of protein A/G-agarose beads. Nonspecifically bound proteins were removed by washing the beads three times with 1 ml of lysis buffer, and bound material was solubilized in 50 µl of 2× Laemmli sample buffer [17], boiled for 5 min, and resolved by SDS-PAGE. The proteins were transferred onto polyvinylidene difluoride membranes, which were blocked for 1 h at room temperature with TBST buffer (20 mM Tris-HCl, pH 7.5, 147 mM NaCl, 0.1% Tween 20) containing 5% nonfat dried milk, and incubated with primary antibody overnight at 4°C. The membranes were then incubated with horseradish peroxidase-conjugated anti-mouse or anti-rabbit secondary antibodies, and the immunoreactive proteins were visualized with an ECL detection system.

### Immunofluorescence staining

Endothelial cells were seeded on 25 mm cover glasses in 6-wells plates and maintained in culture until they reached 60% confluence. Cells were washed with PBS and fixed with 100% methanol for 10 min at −20°C. Non-specific sites were blocked with 2% BSA in PBS for 1 h at room temperature. After being washed, cells were incubated overnight at 4°C with primary anti-STIM1 and anti-IP_3_R-1 antibodies (20 µg/ml) prepared in PBS. After three washes with PBS, cells were incubated for 1 h at room temperature with secondary AlexaFluor 488-conjugated goat antibodies against rabbit IgG (1∶300 dilution) or AlexaFluor 594-conjugated goat antibodies against mouse IgG (1∶300 dilution) (Molecular Probes, Eugene, OR). After extensive washing with PBS, cover glasses were mounted on microscope slides using Vectashield (Vector Laboratories, Burlingame, CA, USA) and examined on a Zeiss Axio Observer microscope (40× oil immersion objective). Pictures were obtained with a Zeiss Axiocam MRm camera using AxioVision LE software. In control experiments performed in parallel, no specific immunofluorescent staining was observed when primary antibodies were omitted.

### Transfection

Six-well plates of BAECs were cultured to 70% of confluence. BAECs were transfected with 40 nM of siRNA using 0.2% of LipofectAMINE 2000 following the protocol provided by the manufacturer (Invitrogen). The cells were maintained in DMEM 10% FBS without antibiotics. The sequences of the sense and anti-sense small interfering RNAs against STIM1 are 5′-CCAAGGAGCACAUGAAGAAdTdT-3′ and 5′-GGUUCCUCGUGUACUUCUUdTdT-3′; and those against STIM2 are 5′-UGACAAAGAUGGUGGAAUCUUdTdT-3′ and 5′-UUACUGUUUCUACCACCUUAGdTdT-3′. Fresh medium was added 5 h post-transfection, and experiments were conducted 48 h after transfection.

### Quantitative PCR

Total RNA was extracted from BAECs, transfected or not with appropriate siRNA oligonucleotide, using TRIzol. RNA quality and presence of contaminating genomic DNA was verified as previously described [18]. RNA integrity was assessed with an Agilent 2100 Bioanalyzer (Agilent Technologies, Mississauga, ON). Reverse transcription was performed on 2.2 µg total RNA with Transcriptor reverse transcriptase, random hexamers, dNTPs (Roche Diagnostics, Laval, QC), and 10 units of RNAseOUT (Invitrogen) following the manufacturer's protocol in a total volume of 20 µl. All forward and reverse primers were individually resuspended to 20–100 µM stock solution in Tris-EDTA buffer (IDT, Coralville, IA) and diluted as a primer pair to 1 µM in RNase DNase-free water (IDT). Quantitative PCR (qPCR) reactions were performed in 10 µl in 96 well plates on a CFX-96 thermocycler (BioRad, Mississauga, ON) with 5 µl of 2× iTaq Universal SYBR Green Supermix (BioRad), 10 ng (3 µl) cDNA, and 200 nM final (2 µl) primer pair solutions. The following cycling conditions were used: 3 min at 95°C; 50 cycles: 15 sec at 95°C, 30 sec at 60°C, 30 sec at 72°C. Relative expression levels were calculated using the qBASE framework [19] and the housekeeping genes ubiquitin (UBC), beta-2-microglobulin (B2M) and Ribosomal protein (RPLP0) for bovine cDNA. Primer design and validation was evaluated as described elsewhere [18]. In every qPCR run, a no-template control was performed for each primer pair and these were consistently negative. All primer sequences are shown in Table 1.

**Table 1:** Primers set used for the quantitative PCR analysis.

Gene	NCBI Accession Number	Foward Primer	Reverse Primer
B2M	NM_173893	5′-GATGGCTCGCTTCGTGGCCT-3′	5′-AGCAGTTCAGGTAATTTGGCTTTCCA-3′
UBC	NM_001206307	5′-GACCGGGAGTTCAGTCTTCGTTC-3′	5′-TCCAGAGTGATGGTTTTACCAGTGAGG-3′
RPLP0	NM_001012682	5′-TGGCAATCCCTGACGCACCG-3′	5′-CACGTTGTCTGCTCCCACAATGA-3′
STIM1	NM_001035409	5′-AGACCTCAATTACCATGACCCAACAG-3′	5′-CACTGCACCACCTCATCCACG-3′
STIM2	XM_002688161	5′-GACAACAATGTCAAAGGAACAACGCT-3′	5′-CCGAACAAAACCACATCCAGCG-3′

### Dynamic video imaging of cytosolic Ca^2+^

BAECs grown on glass coverslips were washed twice with HBSS (120 mM NaCl, 5.3 mM KCl, 0.8 mM MgSO_4_, 5 mM glucose, 1.8 mM CaCl_2_, 20 mM Hepes, pH 7.4) and loaded with 0.4 µM fura 2-AM for 30 min at room temperature in the dark. The cells were then washed and bathed in fresh HBSS for 30 min to ensure complete hydrolysis of the fura-2/AM before placing the coverslip in a circular open-bottom chamber mounted on the stage of a Olympus IX71 microscope (Olympus, Markham, ON, Canada) equipped with a Lambda DG-4 illuminator (Sutter Instrument Company, Novato, CA). Fluorescence from isolated fura-2-loaded cells was monitored by videomicroscopy using 340 nm and 387 nm excitatory wavelengths, and emitted fluorescence was recorded at 510 nm. Images were digitized and analyzed with the MetaFluor software (Universal Imaging Corporation, Downingtown, PA). The data are expressed as the intracellular free Ca^2+^ concentration (nM) calculated from the 340/387 fluorescence ratio according to [20]. All experiments were performed at room temperature.

### Data analysis

All experiments were performed at least three times. Results are expressed as mean ± standard deviation (SD). When needed, the data were analyzed using an analysis of variance (ANOVA), and pairwise comparisons were performed using Dunnet's test. In all cases, results were considered statistically significant when *p*<0.05 (*). The concentration response curve fitting were done using the R statistical software 2.14.1 (R Development Core Team, Vienna, Austria) and the EC50s and maximal responses were expressed as best fit ± standard error (SE).

## Results

### STIM1 and STIM2 are expressed in BAECs and participate to SOCE

**Figure 1:**
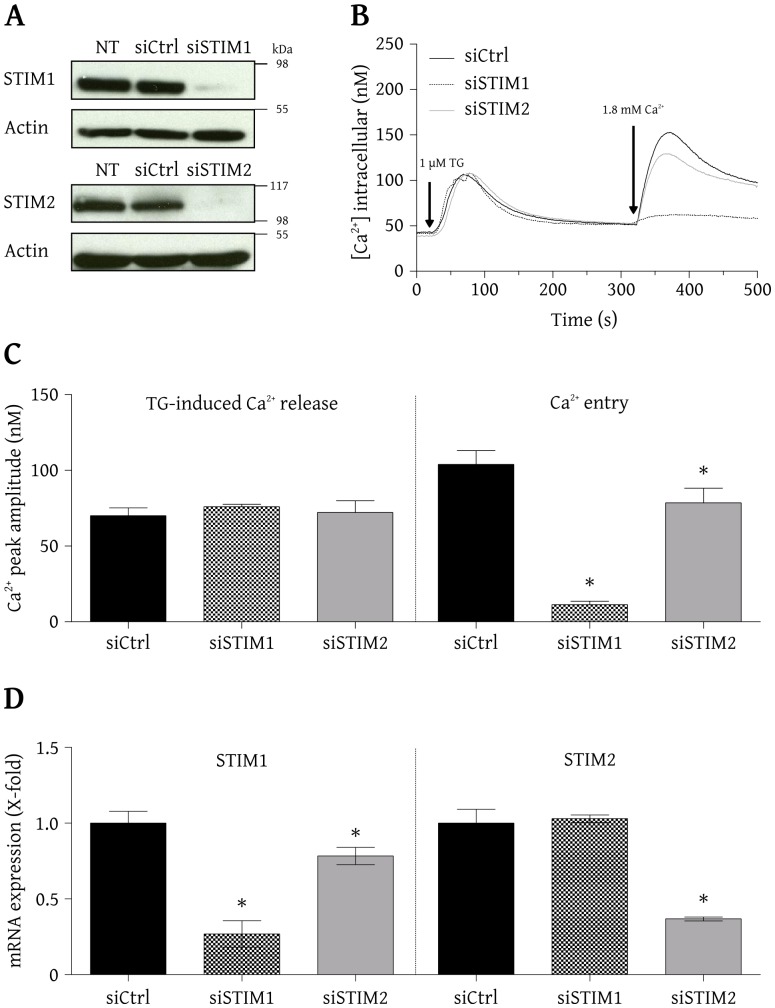
STIM1 and STIM2 are expressed in BAECs and contribute to SOCE. A) Cells were transfected with siCtrl, siSTIM1 or siSTIM2. After 48 h, cells were lysed and proteins were resolved by SDS-PAGE and identified by Western blot using selective antibodies against STIM1, STIM2 or actin (NT indicates non transfected cells). B) BAECs were loaded with fura-2/AM and imaged using an Olympus IX71 microscope (40× oil immersion objective) coupled to a MetaFluor imaging system for the recording of the intracellular Ca^2+^ concentration. In a nominally free Ca^2+^ medium, cells were treated with 1 µM TG to deplete their Ca^2+^ store and, once the Ca^2+^ concentration had stabilized, 1.8 mM Ca^2+^ was added to the medium to induce Ca^2+^ entry. The figure shows average traces from cells (>75 cells/condition) transfected with siCtrl (black line), siSTIM1 (dashed line) or siSTIM2 (gray line). C) Average Ca^2+^ increase (peak amplitude) after treatment with TG and subsequent Ca^2+^ entry (mean ± SD of results obtained from 3–4 independent experiments, *p<0.05). D) Total RNA was extracted from transfected cells (48 h post-transfection) and subjected to a qPCR analysis using specific primers for STIM1 and STIM2 to evaluate their relative level of encoding mRNAs. The results represent the mean ± SD of three independent experiments.

The qPCR analysis shown in Fig. 1D (left) reveals that the relative level of encoding STIM1 mRNA was efficiently lowered in siSTIM1 cells compared to siCtrl cells (0.27±0.09 and 1.00±0.08, respectively) without affecting the levels of STIM2 mRNA (1.03±0.03 in siSTIM1 cells and 1.00±0.09 in siCtrl cells). The relative level of STIM2 mRNA (Fig. 1D, right) was also efficiently decreased in siSTIM2 cells compared to siCtrl cells (0.37±0.01 and 1.00±0.09 respectively). The level of STIM1 mRNA was also slightly reduced in siSTIM2 cells (0.78±0.06 in siSTIM2 cells and 1.00±0.08 in siCtrl cells). Western blots shown in Fig. 1A indicate that under our experimental conditions, the proteins STIM1 (top panel) and STIM2 (bottom panel) are expressed in BAECs. Fig. 1A also shows that the level of STIM1 and STIM2 expression were efficiently (>90%) lowered by their respective siRNAs. Not targeting siRNA (siCtrl) did not alter STIM1 and STIM2 expression. To determine the functional consequence of STIM1 and STIM2 knockdown, the IP_3_-sensitive Ca^2+^ pool content and the activation of SOCE were evaluated in intact BAECs. BAECs were bathed in a nominally Ca^2+^ free medium and treated with 1 µM thapsigargin (a SERCA pump inhibitor). Thapsigargin increased the intracellular Ca^2+^ to a similar level in BAECs transfected with siCtrl, siSTIM1 or siSTIM2 (Fig. 1B). The average peak amplitudes were 70.0±5.2 nM, 76.0±1.6 nM and 72.2±7.7 nM, respectively (Fig. 1C, left). The subsequent addition of 1.8 mM extracellular Ca^2+^ revealed that the SOCE was attenuated in cells transfected with siSTIM1 or siSTIM2, as compared to cells transfected with siCtrl (Fig. 1B). The average peak amplitude was 103.9±9.1 nM in cells transfected with siCtrl and was significantly reduced to 78.6±9.6 nM in cells transfected with siSTIM2 and nearly abolished to 11.3±2.2 nM in cells transfected with siSTIM1 (Fig. 1C, right). It is important to mention that under each condition, the basal intracellular Ca^2+^ concentration was similar. The moderate reduction of STIM1 mRNA expression in siSTIM2 cells presumably contributed to reduce the SOCE in these cells. These results revealed that the knockdown of STIM1 or STIM2 did not alter the content of the IP_3_-sensitive Ca^2+^ pool in BAECs but moderately (for STIM2) or strongly (for STIM1) affected their SOCE activity.

### STIM1 and STIM2 co-immunoprecipitate with IP_3_Rs

**Figure 2:**
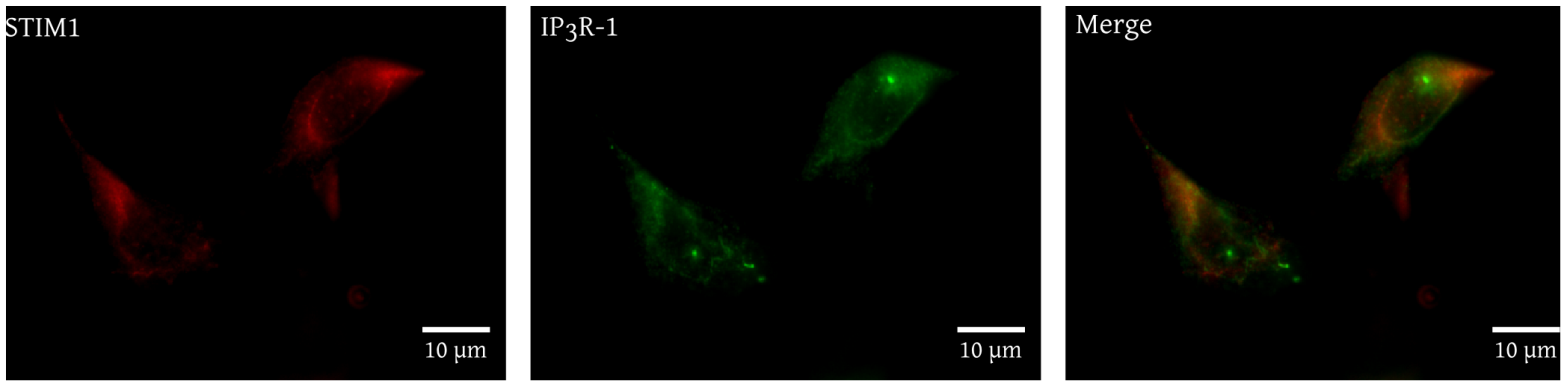
STIM1 and IP_3_R-1 are widely distributed throughout the endoplasmic reticulum in BAECs. A) BAECs were grown on cover glasses, fixed with methanol and incubated with mouse anti-STIM1 and rabbit anti-IP_3_R-1 antibodies. Fluorescent staining was obtained with AlexaFluor 594-conjugated (STIM1, left panel) and AlexaFluor 488-conjugated (IP_3_R-1, center panel) secondary antibodies. The right panel represents the overlay of these images. The results are representative of three independent experiments performed on different cells preparations.

To verify whether STIMs could functionally interact with IP_3_R under basal conditions, we first examined if their intracellular localization made this possible in BAECs. Fig. 2 shows the immunostaining obtained with anti-STIM1 and anti-IP_3_R-1 antibodies in untransfected and unstimulated BAECs. Using the anti-STIM1 antibody, the fluorescence was widely distributed throughout the cell with a higher intensity in the perinuclear region corresponding to the endoplasmic reticulum. The outer limits of the cell were not clearly defined, which indicates that the plasma membrane was not stained (left panel). Similar results were obtained with the anti-IP_3_R-1 antibody (central panel). The overlay image of the two staining clearly shows that STIM1 and IP_3_R-1 were mostly present in the same region of the endoplasmic reticulum and that their physical interaction was possible in a wide part of the cell (right panel). A co-immunoprecipitation approach was used to further verify whether these two proteins interact together. Isoform specific antibodies were used to precipitate the IP_3_R-1 from BAECs lysates and the presence of STIM1 and STIM2 in the resulting immune complex was verified with isoform specific antibodies.

**Figure 3:**
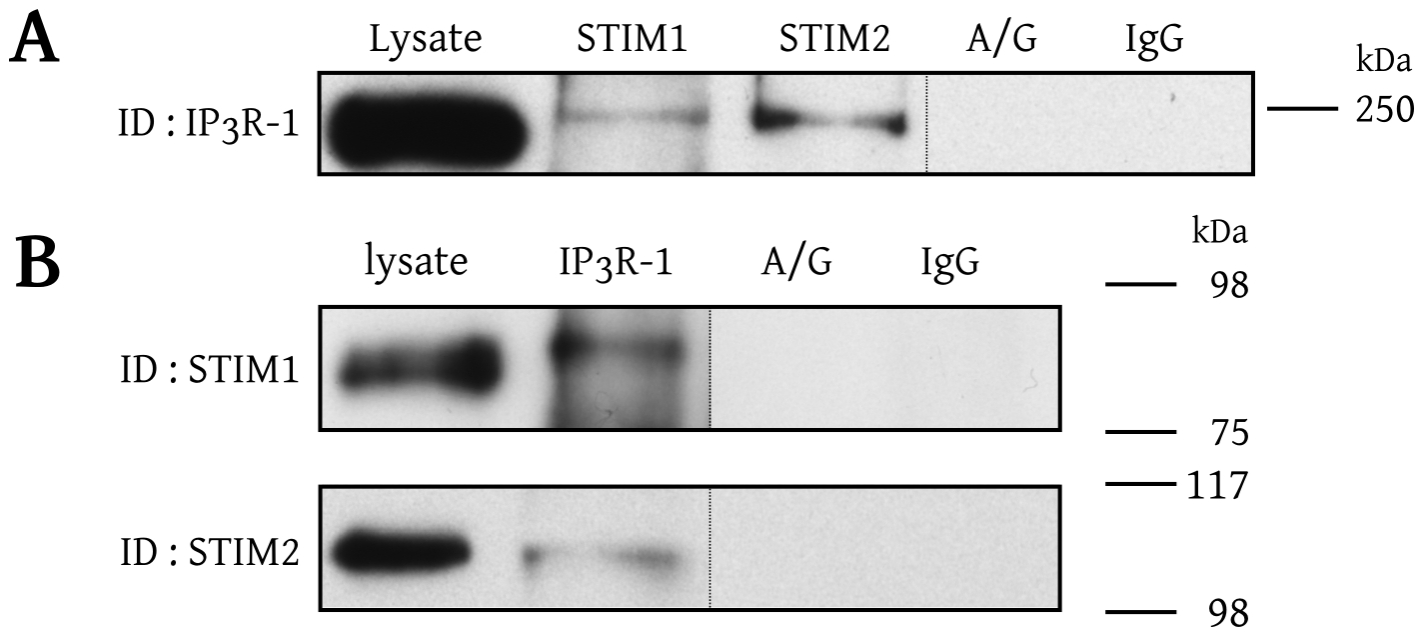
STIM1 and STIM2 interact with IP_3_R-1. A) BAECs were solubilized in 1% Triton X-100 and the lysate was fractionated into samples that were immunoprecipitated with isoform-specific anti-STIM antibodies or, as control conditions, with IgG antibodies (IgG) or exclusively with protein-A/G agarose beads (A/G). The resulting immune complexes were separated by SDS-PAGE, transferred to PVDF membranes, and immunoblotted with an isoform-specific anti-IP_3_R-1 antibody as indicated on the left side of the blot. B) BAECs lysate was immunoprecipitated with anti-IP_3_R-1 antibody and the blot was revealed with an anti-STIM1 or anti-STIM2 antibodies as indicated. These results are representative of at least three independent experiments performed with different cells preparations.

The Western blots showed that both STIM1 and STIM2 interact with IP_3_R-1 (Fig. 3A). Considering the high level of STIM1 and STIM2 detected in the small fraction of BAECs lysates (3% v/v), and the relatively low level of STIM1 and STIM2 detected in the immune complex from the whole lysates, it must be concluded that a very small proportion of STIMs are implicated in these interactions. Nevertheless these results suggest that STIM1 and STIM2 physically interact with IP_3_R-1. To further confirm the presence of a physical interaction between STIMs and IP_3_R-1, BAECs lysates were immunoprecipitated with anti-STIM1 or anti-STIM2 antibodies and IP_3_R-1 was detected in these immunoprecipitates (Fig. 3C).

### The knockdown of STIM1 dampens the IP_3_R-dependent intracellular Ca^2+^ release in BAECs

**Figure 4:**
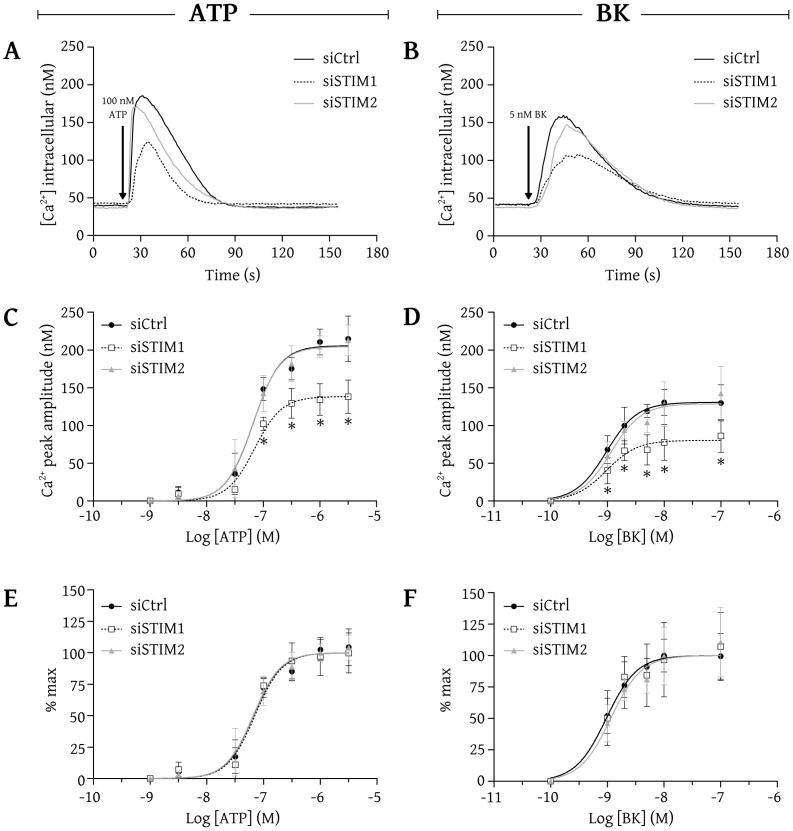
The knockdown of STIM1 dampens the IP_3_R-dependent agonist-induced intracellular Ca^2+^ release in BAECs. BAECs were loaded with fura-2/AM and imaged using an Olympus IX71 microscope (40× oil immersion objective) coupled to a MetaFluor imaging system for the recording of intracellular Ca^2+^ concentration. (A and B) Average traces from cells (>75 cells/condition) transfected with siCtrl (black line), siSTIM1 (dashed line) or siSTIM2 (gray line) stimulated with 100 nM ATP (A) or 5 nM BK (B), in a nominally free Ca^2+^ medium. (C and D) Average Ca^2+^ releases (mean ± SD of results obtained from 4–6 independent experiments) induced by increasing concentrations of ATP (C) or BK (D). (E and F) Same data as in C and D expressed as the percentage of the maximal response under each condition. * indicates that the results are significantly different from those obtained with cells transfected with siCtrl.

We verified whether STIM1 and STIM2 influence the IP_3_R-dependent intracellular Ca^2+^ release in intact BAECs. A videomicroscopic approach was used to monitor the intracellular Ca^2+^ concentration following stimulation with ATP or bradykinin (BK), two well known Ca^2+^-mobilizing agonists in BAECs [21]–[22]. To focus exclusively on IP_3_R-dependent Ca^2+^ release, the experiments were done in a nominally free Ca^2+^ extracellular medium. Fig. 4A shows the integrated Ca^2+^ responses of pre-selected BAECs (average response of 25 cells) transfected with siCtrl (solid black line), siSTIM1 (dashed black line) or siSTIM2 (solid gray line) after stimulation with 100 nM ATP, a submaximal concentration to release Ca^2+^. ATP (100 nM) increased the intracellular Ca^2+^ concentration from around 40 nM to 180 nM in cells transfected with siCtrl, to 125 nM in cells transfected with siSTIM1 and to 171 nM in cells transfected with siSTIM2. A submaximal concentration of BK (5 nM) increased the intracellular Ca^2+^ concentration from around 40 nM to 160 nM in cells transfected with siCtrl, to 106 nM in cells transfected with siSTIM1 and to 147 nM in cells transfected with siSTIM2 (Fig. 4B). These results show that the knockdown of STIM1 significantly reduced the peak amplitude of IP_3_R-dependent Ca^2+^ release whereas the knockdown of STIM2 did not significantly alter IP_3_R-dependent Ca^2+^ release in BAECs.

We repeated these experiments using increasing concentrations of ATP and reported graphically the mean peak amplitude obtained with each concentration, as shown in Fig. 4C. Nonlinear regression analysis furnished the concentration-response curve that best fitted these data, as shown in Fig. 4C. The curves clearly indicate that over the range of concentrations used, the cells transfected with siSTIM2 exhibited an IP_3_R-dependent Ca^2+^ release similar to that of cells transfected with siCtrl. Actually, the two curves are nearly superimposable. However, cells transfected with siSTIM1 showed significantly lower Ca^2+^ responses upon stimulation with high concentrations of ATP (>100 nM). The peak Ca^2+^ response obtained with a maximal concentration of ATP was 206±5 nM Ca^2+^ in cells transfected with siCtrl, 205±5 nM Ca^2+^ in cells transfected with siSTIM2 and 138±4 nM Ca^2+^ (67% of control response) in cells transfected with siSTIM1.

Concentration-response curves were also obtained using BK (Fig. 4D). As observed with ATP, cells transfected with siSTIM2 responded similarly to cells transfected with siCtrl, whereas cells transfected with siSTIM1 had a significantly lower Ca^2+^ response upon stimulation with high concentrations of BK. The peak Ca^2+^ response obtained with a maximal concentration of BK was 131±4 nM Ca^2+^ in cells transfected with siCtrl, 129±6 nM Ca^2+^ in cells transfected with siSTIM2 and 80±5 nM Ca^2+^ (62% of control response) in cells transfected with siSTIM1. These results also show that BK is less efficient than ATP to mobilize Ca^2+^. Indeed, in control cells, the maximal response obtained with BK corresponds to only 64% of the maximal response obtained with ATP. Interestingly, while the maximal response obtained with BK is 36% lower than that obtained with ATP, the reduction of the maximal response of cells transfected with siSTIM1 is similar with both hormones (about 35% lower than that of control cells). To illustrate the effect of the knockdown of STIM1 and STIM2 on the apparent affinities of both agonists, the data shown in Fig.4C and Fig. 4D were expressed as a function of the maximal response obtained under each condition. Fig. 4E and Fig. 4F show that the concentration-response curves nearly superimposed, indicating that the apparent agonist affinities were not affected by the knockdown of STIM1 or STIM2. ATP showed an EC50 of 65±6 nM in cells transfected with siCtrl, of 69±9 nM in cells transfected with siSTIM1 and of 64±6 nM in cells transfected with siSTIM2. BK showed an EC50 of 1.0±0.1 nM in cells transfected with siCtrl, of 1.0±0.2 nM in cells transfected with siSTIM1 and of 1.2±0.2 nM in cells transfected with siSTIM2. These results indicate that the knockdown of STIM1, but not that of STIM2, dampens the IP_3_R-dependent agonist-induced intracellular Ca^2+^ release in BAECs without affecting the apparent affinity of the Ca^2+^-mobilizing agonists.

## Discussion

In endothelial cells, both IP_3_R-dependent Ca^2+^ release and SOCE contribute to shape the agonist-induced Ca^2+^ response [15]. However, most of the work toward the characterization of the role of STIMs in endothelial cells has focused exclusively on Ca^2+^ entry [9]–[14]. In the present study, we evaluated the contribution of STIMs on IP_3_R-dependent Ca^2+^ release. We showed that STIM1 and STIM2 are expressed in BAECs, which is also the case in most cellular types including endothelial cells [9]–[10], [13]–[14], [23]–[24]. We further showed that, without affecting the amount of Ca^2+^ available in the ER, the knockdown of STIM1 nearly abolished the SOCE while the knockdown of STIM2 resulted in a minor reduction of the SOCE. A strong abolition of the SOCE induced by the knockdown of STIM1 has also been reported in human umbilical vein endothelial cells (HUVEC and EA.hy926 cells), in porcine aortic endothelial cells (PAEC) and in mouse lung endothelial cells, thus confirming the crucial role of STIM1 in endothelial SOCE [9]–[11], [14]. To the best of our knowledge, no quantification of the contribution of STIM2 to endothelial SOCE has been reported, probably because of the strong contribution of STIM1 that may mask the weak contribution of STIM2. However, we showed here that STIM2 contributes to a small fraction of SOCE in BAECs in the presence of native STIM1. These results are in accordance with numerous studies addressing the differential roles of STIM1 and STIM2 which, with the exception of rare specific cases, point toward a major role of STIM1 in SOCE (for a review on the subject see [6] and [25]. Some studies reported a role for STIM2 in the regulation of the basal cytosolic Ca^2+^ concentration in different cell types including HUVEC [25]–[26]. In the specific case of HUVEC, the basal cytosolic Ca^2+^ concentration was lowered by about 3 nM after silencing STIM2 [26]. In BAECs, we did not observe any significant change in the basal cytosolic Ca^2+^ level after silencing STIM1 or STIM2.

The activity of IP_3_R is known to be finely tuned by a variety of interacting molecules and by the luminal or cytosolic Ca^2+^ concentration [5], [29]. Since the available Ca^2+^ in the ER and the basal cytosolic Ca^2+^ concentration did not change when STIM1 or STIM2 were silenced, the only way they can modulate the IP_3_R-dependent Ca^2+^ release is via an interaction with a component that leads directly to Ca^2+^ release via IP_3_R.

We showed that IP_3_R-1 and STIM1 share a similar pattern of distribution throughout BAECs, which is a sign of proximity between them. The wide distribution of these two proteins could allow the regulation of the Ca^2+^ release at a cellular scale rather than only in focalized regions of the cells. Using co-immunoprecipitation studies, we demonstrated that STIM1 and STIM2 interact with IP_3_R-1. Another example of proximity between STIMs and IP_3_Rs has been provided in HEK293 cells where the formation of a protein complex composed of Orai1, STIM1, TRPC3, RACK3 and IP_3_R-1 is formed upon activation with ATP or Carbachol [27]. Also, in MDCK cells, an interaction of STIM1 with IP_3_R-3 has been observed upon activation with ATP [28]. More specifically in endothelial cells, STIM1 has been shown to be actively transported by microtubules to the front edge of migrating HUVEC were IP_3_R-mediated Ca^2+^ release are also more frequently observed [29]. Together, these observations suggest that STIMs may interact with IP_3_R.

Our results also revealed that the knockdown of STIM1, but not that of STIM2, dampens the IP_3_R-dependent intracellular Ca^2+^ release induced by ATP and BK in intact BAECs. Similar results were observed in HEK293 cells where the formation of a protein complex composed of Orai1, STIM1, TRPC3, RACK3 and IP_3_R-1 was shown to increase the IP_3_R-dependent Ca^2+^ release [27]. Isolated from STIM1-knockout mice, mast cells stimulated with IgE, embryonic fibroblasts stimulated with ATP and platelets stimulated with ADP, thrombin, or thromboxane A_2_ showed a reduced intracellular Ca^2+^ release [30]–[32]. These studies from independent laboratories suggest a role for STIM1 in the agonist-induced Ca^2+^ release via IP_3_R.

Using concentration-response analyses, we demonstrated that the knockdown of STIM1, but not that of STIM2, reduced the maximal IP_3_R-dependent intracellular Ca^2+^ release in BAECs stimulated with ATP or BK without affecting their apparent affinity. These results suggest that STIM1 exerts a direct effect on IP_3_R functionality. Recently, Santoso et al. [28] suggested a mechanism in which STIM1 binds to IP_3_R-3 to prevent the binding of polycystin-2 in MDCK cells. Located on the ER, polycystin-2 is a Ca^2+^ channel activated by Ca^2+^ that enhances the IP_3_-dependent Ca^2+^ release when interacting with the IP_3_R [28], [33]–[35]. By a similar mechanism, STIM1 could also prevent the inactivation of IP_3_Rs by other molecules. An example of such a molecule is CIB1, a widely expressed Ca^2+^ binding protein, that has been shown to bind and inactivate IP_3_R, thus reducing the number of channels available for activation by IP_3_ [36]. Alternatively, STIM1 may also directly potentiate IP_3_R activity by stabilizing a conformation allowing an increased flux of Ca^2+^ upon activation. Some interacting proteins or kinases have been shown to directly modulate IP_3_R gating by affecting its open probability (for a review see [2]). STIM1 localization and activity were suggested as key features to maintain the spatial and temporal dynamics of the Ca^2+^ signal necessary to promote HUVEC migration [29]. A positive regulatory role of STIM1 on IP_3_R activity is compatible with such a mechanism. Further investigations are needed to establish precisely how STIM1 induces a positive effect on IP_3_R functionality in endothelial cells.

In conclusion, we showed that STIM1 and STIM2 are expressed in BAECs and that SOCE strongly depends on STIM1 and partially on STIM2. We also identified STIM1 and STIM2 as interacting partners for IP_3_R-1, which is a sign of proximity between STIMs and IP_3_R populations. Moreover, we demonstrated that STIM1, but not STIM2, is a positive regulator of IP_3_R in BAECs. The mechanism does not involve a change in the sensitivity of IP_3_R for IP_3,_ but the results rather suggest that STIM1 increases the efficacy of IP_3_R. Therefore, while the role of STIM2 appears to be minor, STIM1 plays an important role in the regulation of agonist-induced Ca^2+^ mobilization in BAECs by a positive effect on both the SOCE and the IP_3_R-dependent Ca^2+^ release.
